# Perceptions of artificial intelligence for radiographic image accuracy: a cross-sectional survey of radiology personnel

**DOI:** 10.3389/fmed.2026.1971679

**Published:** 2026-09-29

**Authors:** Reham Kaifi, Byadr Yamani, Rawan Almarwani, Raghad Qahaf

**Affiliations:** 1 Department of Radiological Sciences, College of Applied Medical Sciences, King Saud bin Abdulaziz University for Health Sciences, Jeddah, Saudi Arabia; 2 Health Affairs, Ministry of the National Guard, Jeddah, Saudi Arabia; 3 King Abdullah International Medical Research Center, Jeddah, Saudi Arabia

**Keywords:** artificial intelligence, attitude of health personnel, diagnostic imaging, machine learning, radiology

## Abstract

**Objectives:**

To evaluate the perceptions of radiology students, interns, and technologists regarding the role of artificial intelligence (AI) in improving the accuracy of radiographic images, and to compare these perceptions across the three groups.

**Materials and methods:**

A quantitative, cross-sectional study was conducted at King Saud bin Abdulaziz University for Health Sciences (KSAU-HS) campuses in Riyadh, Jeddah, and Al-Ahsa, and at National Guard hospitals in Jeddah, between January and December 2025. Radiology students, interns, and technologists were recruited by non-probability convenience sampling (*n* = 81). Data were collected through a structured, self-administered online questionnaire using a five-point Likert scale. Descriptive and inferential statistics (Chi-square, Mann–Whitney U, and Kruskal–Wallis tests) were performed using Microsoft Excel and JMP, with significance set at 0.05.

**Results:**

Of 81 respondents, 91.4% self-rated their AI knowledge as good to excellent, although 60.5% reported never having used AI tools in radiological practice. Most respondents (79.0%) had not received AI-related training, and 86.4% agreed that combining AI with human expertise increases diagnostic accuracy. AI was widely regarded as an effective diagnostic aid (87.7%), although trust in AI-processed radiological images was considerably lower (30.9% agreement, 42.0% neutral). The overall composite AI-attitude score (median 3.85, IQR 3.54–4.15; mean 3.82 ± 0.53) showed no statistically significant differences in this sample across the subgroups examined, including gender (*p* = 0.063), professional role (*p* = 0.193), prior AI use (*p* = 0.086), or formal AI training (*p* = 0.178); effect sizes were small. Most respondents (82.7%) disagreed that AI will replace radiologic technologists.

**Conclusion:**

Participants held positive perceptions of AI as a supportive tool and believed that its integration with human expertise could improve radiographic image accuracy and reduce diagnostic errors, without viewing AI as a replacement for radiologic technologists. These findings reflect participants' perceptions rather than objectively measured diagnostic performance.

**Clinical relevance statement:**

Understanding how radiology students, interns, and technologists perceive artificial intelligence supports its integration into clinical imaging workflows, informs targeted training programs, and helps align professional expectations with the realistic role of AI in improving radiographic image accuracy.

## Graphical Abstract

**Figure:**
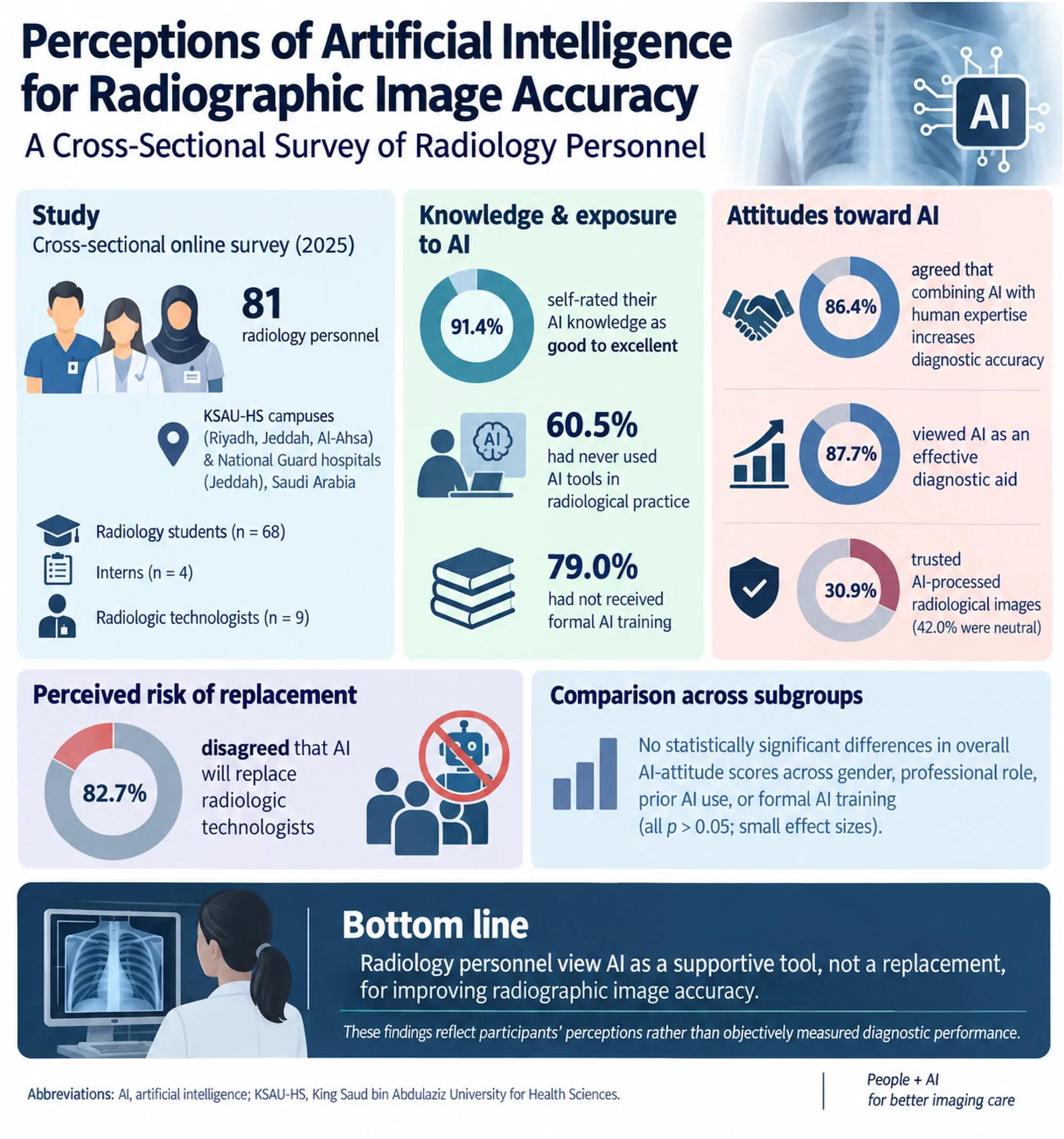

## Highlights

1. Radiology personnel self-rated their knowledge of artificial intelligence (AI) as good to excellent (91.4%), yet routine clinical use remained limited.
2. Overall AI-attitude scores were positive, and no statistically significant differences were detected in this sample by gender, professional role, or training (all *p* > 0.05).
3. Most respondents (82.7%) did not believe AI will replace radiologic technologists.

## Introduction

Artificial Intelligence (AI) technology has become the most debated issue in healthcare, predominantly in medical imaging. AI is defined as the simulation of human-based intelligence in machines designed to adapt and think like humans. It includes the development of computer systems and algorithms capable of performing tasks that generally require human adaptability, reasoning, and mental processes (1). The proficiency of AI in comparing and computing vast imaging datasets has elevated diagnostic accuracy, speed of interpretation, and therapeutic planning. The algorithms incorporated into AI have provided remarkable assistance in detecting malformations, enabling prompt diagnosis of conditions such as cancers and fractures. These algorithms achieved pattern recognition by capturing subtle features often overlooked by humans (2). Disease survival and severity can be predicted using hybrid networks that combine lesion properties with laboratory and clinical data (3, 4). AI has performed well across several clinical imaging tasks, such as image segmentation, predictive analytics, quality control, and personalized treatment planning (1). Although AI is projected to evolve into a standard instrument in the domain, several studies verified that radiographers are not at risk of being excluded, as the technology has only proven its efficacy in specific areas (5–7). Another investigation found that AI is poised to reinforce the function of radiology technologists in medical imaging departments and assist them in performing tasks, such as pre-imaging evaluation, exam planning, and radiographic image analysis (8). In addition, the tool can support alleviating workload pressure, automating routine tasks, and triaging cases (9, 10). In these performance studies, such improvements have been associated with reduced human error and with better patient outcomes and diagnostic accuracy (2). Nevertheless, numerous obstacles exist to the application of AI technology in clinical environments (11). These challenges encompass ethical, medico-legal, and psychological concerns (12, 13). Ethical issues are linked to acquiring informed consent, patient privacy, potential biases in AI algorithms, and responsibilities for faults. Medico-legal considerations pertain to adherence to regulatory requirements and the accountability of AI-produced diagnoses. Perceiving and trusting AI-aided diagnoses are major psychological concerns for both caregivers and patients; both require greater transparency and training when AI tools are integrated into imaging procedures (2). It is critical for healthcare providers to recognize the implications of AI, as well as its relevance and role in advancing radiology (14). Additionally, comprehensive training in the employment of AI is essential for medical practitioners to elevate their trust in it (15). Three related but distinct dimensions determine whether AI delivers benefit in practice: the technical performance of AI systems, the perceptions and acceptance of the healthcare professionals who must use them, and the organizational conditions required for safe and effective implementation. The literature summarized above addresses principally the first of these. Successful adoption, however, depends not only on algorithmic accuracy but also on professional trust, role clarity, education, accountability, governance, and the organizational capacity to integrate AI into routine workflows. The effectiveness of AI in medical imaging is therefore shaped in large part by the preparedness of the radiology workforce to adopt it, including radiology students, interns, and radiologic technologists. It is vital to understand these groups' perceptions of AI deployment to better understand their perspectives toward its adoption in clinical environments and to identify potential obstacles that may impede its application. For this reason, this study aims to evaluate the perceptions of radiology students, interns, and radiologic technologists regarding the role of AI in enhancing the accuracy of radiographic images. For clarity, the following terms are used consistently throughout: “radiology students” denotes undergraduate students enrolled in the radiological sciences programme; “interns” denotes graduates undertaking a supervised clinical internship year; and “radiologic technologists” denotes qualified professionals employed to perform diagnostic imaging examinations. The term “radiographer”, used in several of the international studies cited here, is treated as equivalent to “radiologic technologist”, while recognizing that scopes of practice differ between healthcare systems. Because the perceptions of these groups have not previously been examined in this setting, the study is exploratory in nature, and no formal hypotheses were specified in advance. It addresses the following research questions:

1. How confident are radiology students, interns, and radiologic technologists in implementing AI?
2. How do the perceptions of these three groups compare with respect to the usefulness of AI in enhancing radiographic images?

## Materials and methods

### Study design and population

This was a quantitative, cross-sectional, descriptive study, conducted at KSAU-HS campuses in Riyadh, Jeddah, and Al-Ahsa, and National Guard hospitals in Jeddah, during the period from January to December 2025. The total population included radiology students, interns, and radiologic technologists. Specifically, undergraduate radiological sciences students and interns currently undergoing practical clinical training in radiology at three campuses of KSAU-HS were included as participants. In addition, the study included radiologic technologists working only at National Guard hospitals in Jeddah. Because students and interns were recruited from the three KSAU-HS campuses whereas practising technologists were recruited from a single hospital group in Jeddah, professional role and geographical setting are partially confounded. The resulting groups also differ markedly in size (students *n* = 68, radiologic technologists *n* = 9, interns *n* = 4), and comparisons between professional roles should therefore be interpreted with caution. To clarify, individuals who were not in the radiology specialty were excluded. Furthermore, based on a 5% margin of error and 95% confidence level, the target sample size was 81, calculated using Raosoft (Sample Size Calculator; Raosoft Inc.), drawn from an estimated eligible population of approximately 100 using a non-probability convenience sampling technique. Because conventional sample-size calculations assume probability sampling, this figure represents the number of participants targeted for recruitment rather than a sample that guarantees representativeness of the wider radiology workforce. This study was reported in accordance with the STrengthening the Reporting of OBservational studies in Epidemiology (STROBE) guidelines for cross-sectional studies.

### Data collection

Data for this study were collected at a single point in time using a structured, self-administered questionnaire. The questionnaire was newly developed by the research team for this study, based on the objectives of the research and on a review of the literature on artificial intelligence in medical imaging and radiology, rather than adapted from a previously validated instrument. Content validity was established by computing the Index of Item-Objective Congruence (IOC) from the independent ratings of four subject-matter experts in radiologic sciences, with all items exceeding the accepted threshold of 0.6. Face validity was assessed through a pilot test with a small group of eligible students: prior to full data collection, 20 copies of the questionnaire were pretested with College of Applied Medical Sciences students to assess clarity, relevance, and appropriateness, and internal consistency in the pretest was acceptable (Cronbach's alpha = 0.70). Minor modifications were made to the questionnaire on the basis of the pretest, and the revised instrument was then distributed afresh; the 20 pretest respondents were therefore not included in the final sample of 81. Because the instrument was modified after the pretest, the pretest alpha of 0.70 refers to the pre-modification version of the questionnaire and is not a second measurement of the instrument analysed here. All 13 Likert items were coded uniformly from 1 (strongly disagree) to 5 (strongly agree), and no items were reverse-coded prior to analysis; this includes the single negatively worded item on the eventual replacement of radiologic technologists (Q8), which was entered in its raw direction. There were no missing item responses: all 81 returned questionnaires were complete across the 13 items, so neither imputation nor case exclusion was required. The full questionnaire is available from the corresponding author upon reasonable request. It consisted of four main sections. The first section collected demographic information, including gender, branch of KSAU-HS, current role, and academic year or years of experience. The remaining three sections contained a total of 16 closed questions: three categorical items capturing self-rated general knowledge of AI, prior use of AI tools in radiology, and receipt of formal AI training or lectures; and 13 statements rated on a five-point Likert scale (strongly disagree, disagree, neutral, agree, and strongly agree) covering knowledge of AI, confidence in AI use, and perceptions of the role of AI in improving the accuracy of radiographic images. The 13 Likert items were combined into a composite AI-attitude index for exploratory analysis, while individual item-level results were also reported separately; their full wording is reproduced in Table 1. The online questionnaire was distributed to participants electronically via Google Form, and all participants completed it voluntarily.

**Table 1:** Descriptive statistics for the 13-item AI attitude and knowledge scale (*N* = 81).

Item	Statement	Mean ± SD; Median (IQR)	% Agree/strongly agree	% Neutral	% Disagree/strongly disagree
Q1	Integrating human expertise with AI increases diagnostic accuracy	4.37 ± 0.78; 5.00 (4.00–5.00)	86.4	11.1	2.5
Q2	AI-supported images contribute to more accurate diagnostic decisions	4.21 ± 0.89; 4.00 (4.00–5.00)	82.7	12.3	4.9
Q3	AI can outperform doctors in diagnostic accuracy	2.62 ± 1.14; 2.00 (2.00–3.00)	21.0	28.4	50.6
Q4	I trust the results of AI-processed radiological images	3.04 ± 0.94; 3.00 (2.00–4.00)	30.9	42.0	27.2
Q5	AI can be an effective aiding tool in medical diagnosis	4.23 ± 0.69; 4.00 (4.00–5.00)	87.7	11.1	1.2
Q6	AI is capable of reducing bias and human errors	3.91 ± 0.79; 4.00 (3.00–4.00)	71.6	24.7	3.7
Q7	Human review is necessary if the image is AI-assisted	4.83 ± 0.49; 5.00 (5.00–5.00)	97.5	1.2	1.2
Q8	AI will eventually replace radiologic technologists	1.73 ± 1.18; 1.00 (1.00–2.00)	9.9	7.4	82.7
Q9	AI can enhance the accuracy of radiologic images	4.32 ± 0.70; 4.00 (4.00–5.00)	92.6	6.2	1.2
Q10	AI can help reduce noise in radiographic images	4.31 ± 0.72; 4.00 (4.00–5.00)	91.4	7.4	1.2
Q11	Using AI to improve image accuracy will help reduce diagnostic errors	4.20 ± 0.73; 4.00 (4.00–5.00)	86.4	11.1	2.5
Q12	AI can detect fine details that are difficult for humans to notice	3.80 ± 0.90; 4.00 (3.00–4.00)	64.2	29.6	6.2
Q13	AI can improve accuracy in cases with fine details, e.g., small tumors or fractures	4.09 ± 0.84; 4.00 (4.00–5.00)	80.2	16.0	3.7
	**Overall composite score (13 items)**	**3.82** **±** **0.53; 3.85 (3.54–4.15)**	**—**	**—**	**—**

Response options scored on a 5-point Likert scale (1 = Strongly Disagree to 5 = Strongly Agree). No items were reverse-coded; Q8 is negatively worded and is reported here in its raw direction, so a low mean indicates disagreement with the replacement of radiologic technologists. Medians and interquartile ranges (IQR, reported as the first to third quartile) are given alongside means because the items are ordinal. Percentages are calculated on all 81 respondents; there were no missing item responses. AI, artificial intelligence; SD, standard deviation.

Values in bold indicate the overall composite AI-attitude score (mean of all 13 items).

### Statistical analyses

Statistical analysis was performed using Microsoft Excel and JMP. Descriptive statistics were used to summarize the data and describe the characteristics of the study population. Frequencies and percentages were calculated for categorical variables. Inferential statistics were conducted to examine differences between variables. The Chi-square test was applied for categorical variables. All between-group comparisons of the composite AI-attitude score were made with non-parametric tests, for two reasons stated here once and applied consistently thereafter: the item responses are ordinal, and several of the subgroups compared are very small (interns, *n* = 4; radiologic technologists, *n* = 9; participants reporting formal AI training, *n* = 17), so the assumptions underlying parametric tests cannot be relied upon. The Mann–Whitney *U*-test was used for two-group comparisons and the Kruskal–Wallis test for three-group comparisons. Medians and interquartile ranges were reported as the primary descriptive statistics for subgroup comparisons, alongside means and standard deviations for descriptive purposes. Effect sizes were reported for each comparison, using rank-biserial r for Mann–Whitney *U*-tests and epsilon-squared for Kruskal–Wallis tests, together with 95% confidence intervals for subgroup means as supplementary descriptive information. All statistical tests were performed at a significance level of 0.05. A composite AI-attitude score was calculated as the unweighted mean of the 13 Likert-scale items addressing self-rated knowledge, confidence, trust, and perceived usefulness of AI, all entered on the same 1–5 metric with no items reverse-coded. Although the items span several related constructs, they were summarized in a single exploratory composite index because they were administered on the same response metric and demonstrated good internal consistency in this sample. Individual items were also reported separately to preserve construct-specific interpretation. Internal consistency was assessed using Cronbach's alpha and is reported in Table 2 as evidence of internal consistency in this sample only. The instrument was not formally validated, and no factor-analytic or other construct-validity evidence is claimed. Six subgroup comparisons were performed on the composite score (gender, professional role, branch, self-rated AI knowledge, prior AI use, and formal AI training) with no adjustment for multiplicity; a Bonferroni correction across these six tests would set the threshold at 0.0083. All subgroup comparisons are therefore exploratory, and the resulting *p*-values should be interpreted accordingly rather than as confirmatory tests.

**Table 2:** Internal consistency and distribution of the composite AI-attitude score.

Statistic	Value
Number of items (*k*)	13
Cronbach's alpha	0.872
Item-total correlation range	0.25–0.70
Composite score, mean ± SD; median (IQR)	3.82 ± 0.53; 3.85 (3.54–4.15)
Composite score skewness	−0.09
Composite score excess kurtosis	−0.32
Jarque–Bera statistic (composite)	0.57
Jarque–Bera *p*-value (composite)	0.799
Normality of composite score (*α* = 0.05)	Not rejected (approximately normal)
Individual-item normality (Jarque–Bera, α = 0.05)	Rejected for 8 of 13 items
Coding direction	All 13 items scored 1–5; no items reverse-coded (including Q8)
Cronbach's alpha, Q8 reverse-coded (*k* = 13)	0.789
Cronbach's alpha, Q8 excluded (*k* = 12)	0.873
Corrected item-total correlation, Q8 entered raw	0.45

*C*ronbach's alpha calculated across the 13-item scale in the main sample of 81 respondents, indicating good internal consistency in this sample (α > 0.80); all α values in this table, including the sensitivity analyses below, relate to the main sample and not to the pretest value of 0.70, which was obtained from 20 pretest respondents on the pre-modification version of the questionnaire. Internal consistency alone does not establish construct validity, and the instrument was not formally validated. The reported α of 0.872 corresponds to the specification actually used, in which all 13 items were entered on the 1–5 metric with no items reverse-coded. Because Q8 is negatively worded, a sensitivity analysis is shown: reverse-coding Q8 lowers α to 0.789, whereas excluding Q8 raises it to 0.873. The positive corrected item-total correlation of Q8 in its raw direction (0.45) is consistent with a uniform response tendency across items rather than with a single coherent underlying attitude, and item-level results are therefore reported alongside the composite throughout. Between-group comparisons used non-parametric tests (Mann–Whitney U, Kruskal–Wallis) for the reasons given in the Methods. SD, standard deviation.

### Ethical considerations

Ethical approval was obtained from the Institutional Review Board prior to data collection. All participants provided written informed consent electronically before questionnaire distribution. Participant confidentiality was strictly maintained, with anonymized data stored in password-protected files accessible only to the research team.

## Results

### Results related to descriptive statistics

Of approximately 100 eligible individuals invited to participate, 81 completed the survey, corresponding to an apparent response rate of 81.0%. The sample was heavily weighted toward students, who accounted for 84.0% of respondents, compared with 4.9% interns and 11.1% practicing radiologic technologists; comparisons between professional groups should be read in the light of this imbalance. As summarized in Table 3, the demographic characteristics of the participants include males (50.6%) and females (49.4%), and participants from three regions: Riyadh (22.2%), Jeddah (49.4%), and Al-Ahsa (28.4%). Also, the population distribution by role includes radiologic technologists (11.1%), interns (4.9%), and students (84.0%). Regarding experience levels, the majority of participants were second-year, third-year, fourth-year, and fifth-year students (88.9%). The rest were grouped by experience level: those with 1–5 years, those with more than 5 years, and participants with no prior experience. All participants met the inclusion criteria.

**Table 3:** Demographic and practice characteristics of study participants (*N* = 81).

Characteristic	*n*	%
Gender		
Male	41	50.6
Female	40	49.4
Professional role		
Student	68	84.0
Radiologic technologist	9	11.1
Intern	4	4.9
Branch/campus		
Jeddah	40	49.4
Al-Ahsa	23	28.4
Riyadh	18	22.2
Academic year/experience level		
2nd-year student	7	8.7
3rd-year student	32	39.5
4th-year student	29	35.8
5th-year student	4	4.9
1–5 years' experience	5	6.2
>5 years' experience	1	1.2
No prior experience	3	3.7
Self-rated general AI knowledge		
Excellent	13	16.0
Good	61	75.3
Weak	7	8.7
Prior use of AI tools in radiology		
Yes	32	39.5
No	49	60.5
Received formal AI training/lectures		
Yes	17	21.0
No	64	79.0

AI, artificial intelligence.

Tables 1, 3 show that most respondents self-rated their AI knowledge as good-to-excellent (91.4%). This figure reflects perceived rather than objectively assessed knowledge, as no knowledge test was administered. This high self-rating contrasted with limited practical exposure: 60.5% had never used AI tools in radiology and 79.0% had not received formal training courses on AI applications in radiology. The gap between self-perceived knowledge and actual exposure is one of the more notable findings of this survey and may indicate a shortfall between general awareness of AI and practical preparedness for its clinical use. A high proportion of participants (86.4%) agreed that using AI to improve image accuracy would help reduce diagnostic errors. Regarding their perception of AI-enhanced image support, 82.7% agreed that it leads to more accurate diagnostic decisions, a high proportion. Agreement that AI could outperform physicians in diagnostic accuracy was low overall, with 21.0% agreeing and 50.6% disagreeing. Group-level distributions and *post hoc* comparisons between professional roles were not conducted for this item, so no conclusion is drawn regarding differences between students, interns, and technologists.

The aim was to compare the perceptions of the three groups regarding AI's effectiveness in improving radiographic images. As shown in Table 1 and Figure 1, overall, the respondents' perceptions toward AI were generally positive. Trust in AI-processed radiological images was considerably more reserved than perceived usefulness: only 30.9% agreed that they trusted such images, while 42.0% were neutral and 27.2% disagreed, compared with 87.7% who considered AI an effective aiding tool in medical diagnosis. Respondents therefore appear to endorse AI as an adjunct while remaining cautious about relying on AI-generated output without professional verification. A limited proportion of participants did not agree that AI can reduce bias and human error (3.7%). The item on human oversight produced the strongest consensus in the survey: 97.5% agreed that human review is necessary when an image is AI-assisted, with only 1.2% disagreeing. This result is best read as evidence of support for continued human oversight rather than as a general positive attitude toward AI.

**Figure 1:**
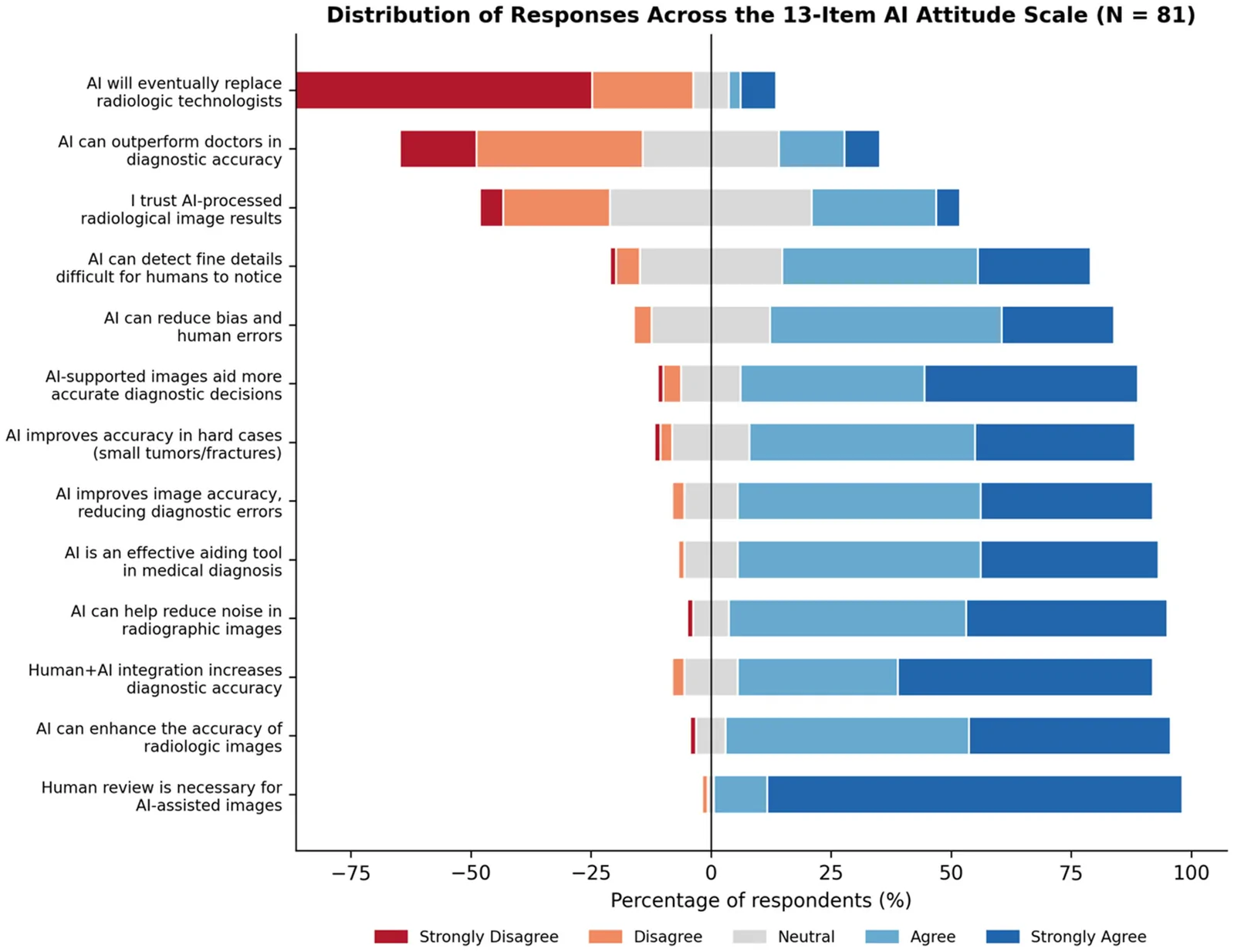
Distribution of responses across the 13-item AI attitude scale (*N* = 81).

The aim was to evaluate the confidence level of radiology students, interns, and technologists in using AI. Table 1 shows that the majority of participants demonstrated a high level of positive perceptions toward the application of AI in radiologic imaging. Figure 2 shows that more than 60% of respondents agreed with AI's ability to enhance image accuracy, reduce radiographic noise, and detect subtle details that may be challenging for human observers. Furthermore, a high proportion of them thought that utilizing AI to improve image quality could contribute to reducing diagnostic errors, particularly in complex cases where fine details, such as small tumors or fractures, are difficult to distinguish.

**Figure 2:**
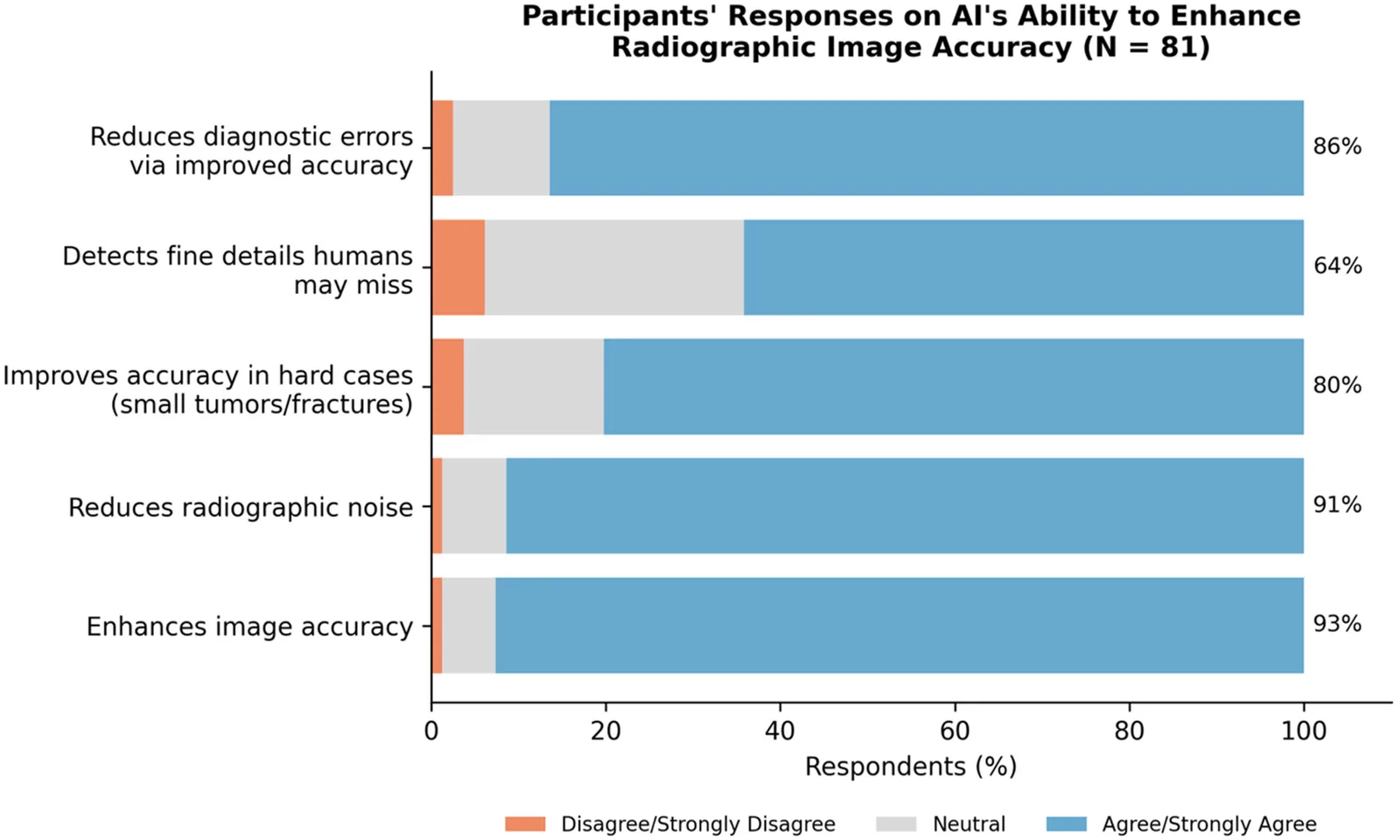
Participants' Responses on AI's ability to enhance radiographic image accuracy (*N* = 81).

Across the whole sample, the composite AI-attitude score was 3.82 ± 0.53 (median 3.85, IQR 3.54–4.15; 95% CI 3.70–3.94), indicating broadly favourable attitudes. Table 4 compares this composite score across participant subgroups. The difference between male participants (mean 3.93 ± 0.52; median 3.85, IQR 3.69–4.23; 95% CI 3.77–4.09) and female participants (mean 3.71 ± 0.54; median 3.69, IQR 3.31–4.10; 95% CI 3.54–3.88) approached, but did not reach, the conventional threshold for statistical significance (Mann–Whitney *U* = 623.50, *z* = 1.86, *p* = 0.063, rank-biserial *r* = 0.240). This difference is described here as an exploratory trend; it is not evidence of a gender effect.

**Table 4:** Comparison of the composite AI-attitude score across participant subgroups.

Subgroup	*n*	Composite score, mean ± SD; median (IQR) [95% CI]	Test	Test statistic; effect size	*P*-value
Gender					
Male	41	3.93 ± 0.52; 3.85 (3.69–4.23) [3.77–4.09]	Mann–Whitney U	U = 623.50, z = 1.86; r = 0.240	0.063
Female	40	3.71 ± 0.54; 3.69 (3.31–4.10) [3.54–3.88]			
Professional role					
Student	68	3.79 ± 0.51; 3.85 (3.48–4.15) [3.67–3.91]	Kruskal–Wallis	H = 3.30, df = 2; *ε*² = 0.041	0.193
Radiologic technologist	9	3.90 ± 0.71; 3.69 (3.54–4.38) [3.35–4.45]			
Intern	4	4.23 ± 0.32; 4.23 (4.08–4.38) [3.72–4.74]			
Branch					
Riyadh	18	3.81 ± 0.49; 3.77 (3.54–4.12) [3.57–4.05]	Kruskal–Wallis	H = 1.26, df = 2; ε² = 0.016	0.534
Jeddah	40	3.87 ± 0.58; 3.88 (3.60–4.31) [3.69–4.06]			
Al-Ahsa	23	3.73 ± 0.49; 3.77 (3.46–4.04) [3.52–3.94]			
Prior use of AI tools					
Yes	32	3.96 ± 0.57; 3.92 (3.62–4.27) [3.75–4.17]	Mann–Whitney U	U = 606.50, z = 1.72; r = 0.226	0.086
No	49	3.73 ± 0.49; 3.77 (3.31–4.08) [3.59–3.87]			
Received formal AI training					
Yes	17	3.97 ± 0.60; 4.08 (3.62–4.54) [3.66–4.28]	Mann–Whitney U	U = 428.00, z = 1.35; r = 0.213	0.178
No	64	3.78 ± 0.51; 3.77 (3.54–4.15) [3.65–3.91]			
Self-rated general AI knowledge					
Excellent	13	3.77 ± 0.65; 3.92 (3.08–4.15) [3.38–4.16]	Kruskal–Wallis	H = 5.11, df = 2; ε² = 0.064	0.078
Good	61	3.78 ± 0.50; 3.77 (3.54–4.15) [3.65–3.91]			
Weak	7	4.24 ± 0.51; 4.23 (4.08–4.58) [3.77–4.71]			

Composite = mean across all 13 scale items (range 1–5), with no items reverse-coded. Between-group comparisons used the Mann–Whitney *U*-test (two groups) or Kruskal–Wallis test (three groups) for the reasons given in the Methods. IQR is reported as the first to third quartile; 95% CIs for group means are t-based and are supplementary to the medians, which are the primary descriptive statistics. Effect sizes are rank-biserial r for Mann–Whitney U (positive values indicate a higher rank sum in the first group listed) and epsilon-squared for Kruskal–Wallis. Six comparisons were performed on the composite score with no adjustment for multiplicity; the Bonferroni-adjusted threshold would be 0.0083. No subgroup comparison reached statistical significance at α = 0.05, which indicates that no differences were detected in this sample rather than that the subgroups held equivalent perceptions; differences should be interpreted as exploratory trends, particularly for small subgroups (Intern, *n* = 4; AI training-yes, *n* = 17; Weak knowledge, *n* = 7). AI, artificial intelligence; CI, confidence interval; IQR, interquartile range; SD, standard deviation.

No statistically significant differences were detected in this sample by professional role (Kruskal–Wallis *H* = 3.30, df = 2, *p* = 0.193, epsilon-squared = 0.041; students median 3.85, IQR 3.48–4.15; interns median 4.23, IQR 4.08–4.38; radiologic technologists median 3.69, IQR 3.54–4.38), branch (*H* = 1.26, df = 2, *p* = 0.534, epsilon-squared = 0.016; Riyadh median 3.77, IQR 3.54–4.12; Jeddah median 3.88, IQR 3.60–4.31; Al-Ahsa median 3.77, IQR 3.46–4.04), or self-rated AI knowledge (*H* = 5.11, df = 2, *p* = 0.078, epsilon-squared = 0.064; excellent median 3.92, IQR 3.08–4.15; good median 3.77, IQR 3.54–4.15; weak median 4.23, IQR 4.08–4.58). The seven participants who rated their AI knowledge as weak recorded the highest mean composite score of any subgroup (4.24 ± 0.51, 95% CI 3.77–4.71), but this rests on seven observations in a comparison that did not reach significance and is reported as an exploratory trend only.

Participants reporting prior use of AI tools (mean 3.96 ± 0.57; median 3.92, IQR 3.62–4.27; 95% CI 3.75–4.17) recorded numerically higher composite scores than those reporting no prior use (mean 3.73 ± 0.49; median 3.77, IQR 3.31–4.08; 95% CI 3.59–3.87; *U* = 606.50, *z* = 1.72, *p* = 0.086, rank-biserial *r* = 0.226), as did those who had received formal AI training (mean 3.97 ± 0.60; median 4.08, IQR 3.62–4.54; 95% CI 3.66–4.28) relative to those who had not (mean 3.78 ± 0.51; median 3.77, IQR 3.54–4.15; 95% CI 3.65–3.91; *U* = 428.00, *z* = 1.35, *p* = 0.178, rank-biserial *r* = 0.213). Neither comparison reached statistical significance, and both should be regarded as exploratory trends rather than as evidence of an association. No subgroup comparison reached statistical significance at *α* = 0.05, indicating that no differences were detected in this sample rather than that the subgroups held equivalent perceptions.

As shown in Figure 3, most participants disagreed with the statement that AI will eventually replace radiologic technologists (82.7%); only a few agreed with this statement.

**Figure 3:**
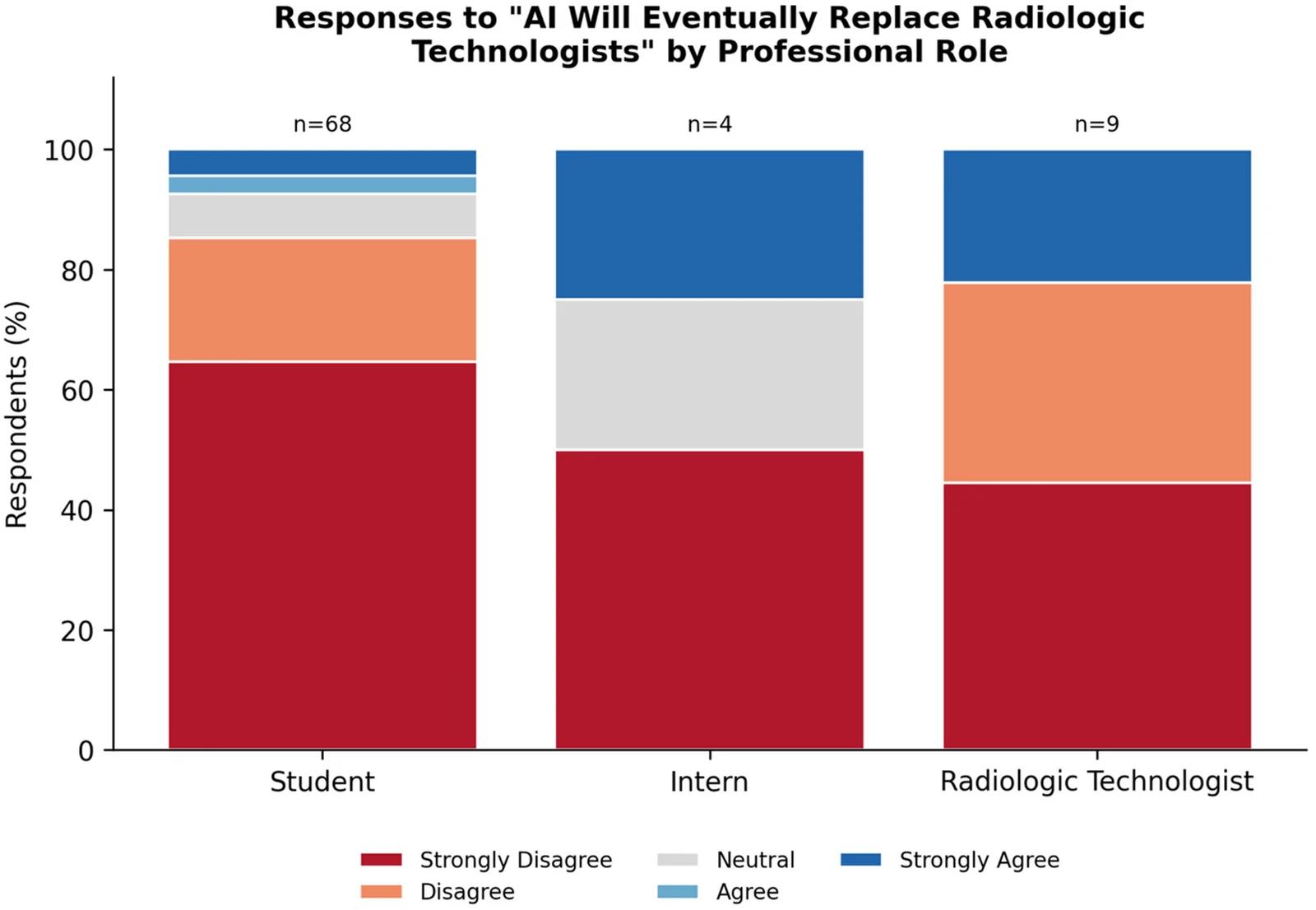
Responses to the statement “AI Will Eventually Replace Radiologic Technologists” by professional role.

**Figure 4:**
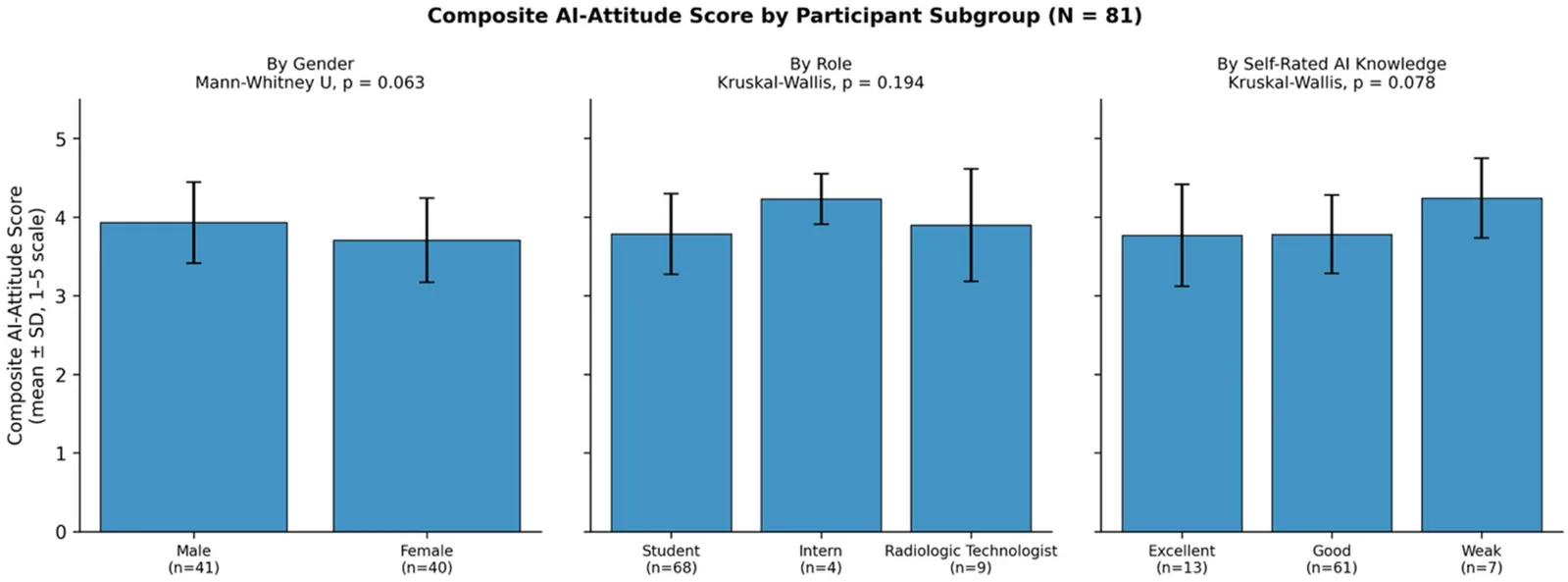
Composite AI-attitude score by participant subgroup (*N* = 81).

### Results related to inferential statistics

Inferential analysis compared the composite AI-attitude score across demographic subgroups (Table 4; Figure 4). Non-parametric tests were applied for the reasons set out in the Methods. No subgroup comparison reached statistical significance at *α* = 0.05; the smallest *p*-value was for gender (*U* = 623.50, *z* = 1.86, *p* = 0.063, rank-biserial *r* = 0.240). Because six comparisons were performed on the composite score without adjustment for multiplicity, and because the effect sizes were small (epsilon-squared 0.016–0.064; rank-biserial r 0.213–0.240), between-group differences should be interpreted as exploratory trends rather than as significant associations. The absence of statistical significance does not establish that the subgroups hold equivalent perceptions; with only four interns and nine radiologic technologists, the study has very low power to detect differences of practical importance.

## Discussion

Recently, AI has shown considerable promise in diagnostic imaging (14); therefore, this study was conducted to assess the perceptions of radiology students, interns, and radiologic technologists toward AI implementation in optimizing radiographic images. Throughout the discussion that follows, we distinguish between evidence of AI performance reported in previous studies and the perceptions measured in the present survey: the findings reported here describe what participants believed AI could achieve, not measured changes in image accuracy or diagnostic outcomes.

Collectively, 91.4% of the participants self-rated their AI knowledge as good-to-excellent. Similarly, a study conducted in 2021 showed that 93% of Spanish medical students documented their knowledge of AI involvement in everyday life (15). In addition, 70.7% correctly answered questions involving basic knowledge of AI. In contrast, a study performed in Germany stated that 68% of participants were unaware of the technologies implemented in the radiological domain (16). The findings of this report represent good awareness and knowledge among participants, similar to studies in different areas, while contrasting with others. These discrepancies may be due to the rising recognition of the involvement of AI in daily life practices, as well as the variations in awareness and knowledge across distinct territories.

In line with the study objectives, the confidence in AI use was evaluated among participants. Most respondents showed neutral attitudes toward trusting AI-generated results. Limited practical exposure or uncertainty about AI outputs may explain this, although this interpretation remains speculative: the questionnaire did not include direct measures of perceived risk, transparency, explainability, or prior hands-on experience with specific AI tools. Conversely, another finding reported that the trust level was relatively limited among medical professionals regarding the reliance on AI for decision-making purposes. A possible cause of this outcome might be connected with concerns regarding the patient's own danger in terms of AI-based assistance (14). This finding is similar to what was stated in the literature, where the rising medico-legal, psychological, and ethical considerations of AI could present a threat to its integrity (12, 13). Moreover, the findings revealed that most participants disagreed when they were asked if AI might ultimately substitute radiographers. The disagreement with this statement may reflect confidence in the persistent role of radiologic technologists regardless of the growing integration of AI in the field, although the survey did not ask participants to explain their reasoning. This aligns with outcomes observed in prior investigations, where a large proportion of participants disagreed with the statement, and only a minority of them agreed (14, 17). However, findings of other reports highlighted different outcomes. An investigation demonstrated that a higher-than-average proportion of respondents agreed (2). Additionally, participants stated that a reduction of radiological staffing levels and the development of new roles are anticipated with the application of AI (18). These varied outcomes suggest that AI adoption is considered distinctly based on workforce preparedness and understanding of its role in clinical practice.

Taken together, the pattern of responses suggests conditional rather than unqualified acceptance of AI. Participants strongly endorsed AI as an aiding tool (87.7%) and supported the combination of AI with human expertise (86.4%), yet fewer than one in three expressed trust in AI-processed images (30.9%) and 97.5% considered human review necessary when an image is AI-assisted. AI therefore appears to be viewed as useful when embedded within a supervised workflow, but not as an autonomous decision-maker, an interpretation that is consistent with the 82.7% who rejected the prospect of AI replacing radiologic technologists. The near-unanimity on human review is what makes the usefulness-trust gap interpretable: the low trust figure is better read as a condition attached to acceptance, namely that AI output be verified by a professional, than as scepticism about the value of AI itself. On that reading the 87.7% and the 30.9% are not in tension, because participants were endorsing AI in a supervised role rather than as a substitute for professional judgement. The practical implication is that acceptance of AI in this setting is likely to depend on the visibility and reliability of the human-review step in the workflow, a proposition this survey raises but cannot test.

Consistent with the aim of this study, subjects' perceptions regarding the AI role in enhancing radiographic images were compared. The vast majority of participants agreed with the statement that AI can elevate radiological image accuracy. Agreement was observed across all three professional groups; however, given the small number of interns (*n* = 4) and technologists (*n* = 9) and the absence of *post hoc* group comparisons, no ranking between the professional roles is inferred. Another report conducted on MRI technologists had similar findings, in which the majority agreed that AI will contribute substantially to enhancing the future of the radiological domain (19). The outcomes presented indicate that participants perceived AI as supportive of the acquisition and interpretation of imaging data; they do not themselves demonstrate that such support was delivered, since no measure of image accuracy or diagnostic performance was obtained in this study. As found in previous studies, the technology has successfully proved its ability to assist in spotting minor discrepancies and abnormalities, improving the analysis of images, minimizing human errors, and thus maintaining high and clinically appropriate diagnostic precision in detecting diseases (20, 21).

This study points to the changing roles of radiologic technologists. This emphasizes the transition from a conventional interpretation of images to a more flexible model in which healthcare professionals must understand and adapt to AI to improve patient care. The combination of high self-rated knowledge with limited practical use and limited formal training may reflect general familiarity with AI rather than preparedness for clinical implementation. A survey of Italian radiographers reported a comparable profile, describing a workforce that was highly familiar with AI in everyday life and showed almost no cultural resistance to it, while professional training and organizational preparation lagged behind that receptiveness (22). These findings therefore support the development of structured educational programmes covering AI fundamentals and limitations, algorithmic bias, clinical validation, data privacy, accountability, human oversight, and workflow integration. Education alone, however, is unlikely to be sufficient: work on organizational health literacy in the transposition of Directive 2013/59/Euratom argues that reorienting radiology departments depends on departmental culture, communication, and governance arrangements as much as on the competence of individual staff (23). That analysis concerns radiation-protection governance rather than AI, and is cited here as an organizational-implementation perspective rather than as evidence about AI; the parallel is nonetheless instructive, since both cases require a department to absorb an externally driven change in practice. Examining these groups' perceptions provided insight into how the radiological profession is changing and how AI is being integrated into it. These findings clarify the continuing role of radiographers as this technology develops.

Despite its contributions, this study has several limitations that should be acknowledged. First, this was a single-institutional study conducted within KSAU-HS and its affiliated National Guard Hospitals, in which the sample size was limited and the results might not be generalizable to other settings. In addition, the voluntary, non-probability convenience sampling approach may have introduced self-selection bias, as individuals with greater pre-existing interest in AI may have been more likely to participate. This may have contributed to the high proportion of respondents reporting good-to-excellent self-rated AI knowledge and generally positive attitudes toward AI. Second, the report predominantly depended on self-reported data, which could lead to bias and may not precisely reveal the respondents' factual knowledge and perception. Third, the cross-sectional design captures participants' perceptions at a single point in time and does not elucidate shifts in attitudes over time. Fourth, the study measured perceptions and self-reported exposure only; it did not assess organizational readiness, implementation capacity, or clinical competence, and the findings should not be read as evidence of institutional readiness for AI deployment. Fifth, although effect sizes and 95% confidence intervals are now reported, no adjustment was made for the six subgroup comparisons performed on the composite score (a Bonferroni threshold would be 0.0083), so the subgroup analyses should be regarded as exploratory and none should be read as confirmatory. Sixth, the negatively worded replacement item was not reverse-coded before being entered into the composite AI-attitude score. Reverse-coding that item lowers the internal consistency of the 13-item scale from *α* = 0.872 to *α* = 0.789, while excluding it raises *α* to 0.873, which suggests that the composite partly reflects a uniform tendency to agree with positively worded statements rather than a single coherent attitude. Item-level results are therefore reported alongside the composite throughout, and the composite should be interpreted with this coding decision in mind. Seventh, the small sizes of several subgroups, particularly interns (*n* = 4), radiologic technologists (*n* = 9), participants with formal AI training (*n* = 17), and those reporting weak self-rated AI knowledge (*n* = 7), substantially limited the statistical power of subgroup comparisons. Therefore, the absence of statistically significant differences should not be interpreted as evidence of equivalent perceptions across groups, as potentially meaningful differences may not have been detected. Additional research with an expanded sample size is crucial to obtain a thorough understanding of AI education and perceptions in the radiological field.

## Conclusion

This study offers insight into how radiology students, interns, and radiologic technologists perceive AI and its applications in radiology. Participants viewed AI favourably as a supportive tool and believed that it could improve radiographic image accuracy, while expressing reservations about trusting AI-processed images without human review and rejecting the prospect of professional replacement. Because the study measured perceptions within a single-institution convenience sample, it does not establish that AI improves image accuracy or diagnostic outcomes, and considerable scope remains for improvement in practical exposure, training, and confidence. Future longitudinal research with an extensive sample size is critical to obtain a comprehensive understanding of the perceptions of AI and to track its progress in radiology.

## Data Availability

The raw data supporting the conclusions of this article will be made available by the authors, without undue reservation.
